# Supplementary material for: Effects of Reduced Amino Acids and Apparent Metabolizable Energy on Meat Processing, Internal Organ Development, and Economic Returns of Cobb 700 and Ross 708 Broilers
Source: Animals (Basel). 2025 Apr 6;15(7):1064. doi: 10.3390/ani15071064 (PMC11988182; doi:10.3390/ani15071064)
Supplement: Supplementary file 1 [file animals-15-01064-s001.zip › animals-3503283-supplementary.pdf]

Supplementary Materials

Table S 1 Feed ingredient and nutrient composition of 12 dietary treatments with factorial combinations of 4 levels of digestible amino acid and 3 levels of apparent metabolizable energy during starter (d 0-10) feeding phase

**Parameter**	**Treatment**											
AA (%)[1]	70	70	70	80	80	80	90	90	90	100	100	100
AME (%)	84	92	100	84	92	100	84	92	100	84	92	100
Yellow Corn %	56.96	67.91	71.06	57.32	64.14	63.94	52.05	60.38	56.74	48.28	55.10	49.55
Soybean Meal %	29.90	22.74	22.27	29.38	28.24	28.27	35.14	33.74	34.35	40.64	39.50	40.43
Soybean oil %	0.00	0.50	2.21	0.00	0.50	3.35	0.50	0.50	4.49	0.50	1.01	5.64
DL-Methionine %	0.18	0.23	0.23	0.29	0.28	0.28	0.33	0.32	0.33	0.38	0.37	0.38
L-Lysine HCl %	0.03	0.23	0.24	0.21	0.23	0.23	0.20	0.22	0.21	0.19	0.21	0.20
L-Threonine %	0.00	0.09	0.09	0.09	0.10	0.10	0.10	0.10	0.10	0.11	0.11	0.11
Ronozyme %	0.02	0.02	0.02	0.02	0.02	0.02	0.02	0.02	0.02	0.02	0.02	0.02
Dicalcium Phosphate %	1.74	1.75	1.74	1.74	1.73	1.73	1.72	1.71	1.72	1.70	1.69	1.70
Limestone %	1.45	1.47	1.48	1.44	1.45	1.45	1.42	1.44	1.43	1.41	1.42	1.41
Salt %	0.40	0.33	0.33	0.33	0.33	0.33	0.33	0.32	0.33	0.33	0.32	0.33
Premix[2] %	0.25	0.25	0.25	0.25	0.25	0.25	0.25	0.25	0.25	0.25	0.25	0.25
Choline chloride %	0.06	0.09	0.09	0.06	0.06	0.06	0.03	0.03	0.03	0.00	0.00	0.00
Sand %	9.00	4.39	0.00	8.87	2.68	0.00	7.90	0.96	0.00	6.19	0.00	0.00
Feed price ($/kg)	0.24	0.24	0.26	0.25	0.26	0.28	0.26	0.27	0.29	0.27	0.28	0.31
Calculated composition												
CP, %	18.70	16.46	16.49	18.77	18.79	18.79	21.10	21.11	21.11	23.42	23.44	23.43
Ca, %	0.96	0.96	0.96	0.96	0.96	0.96	0.96	0.96	0.96	0.96	0.96	0.96
Available *P* %	0.48	0.48	0.48	0.48	0.48	0.48	0.48	0.48	0.48	0.48	0.48	0.48
M.E. (kcal/kg)	2550	2792	3035	2549	2792	3035	2549	2792	3035	2549	2792	3035
Digestible Met %	0.44	0.46	0.46	0.54	0.53	0.53	0.61	0.60	0.61	0.68	0.68	0.68
Digestible TSAA %	0.66	0.66	0.66	0.76	0.76	0.76	0.85	0.85	0.85	0.95	0.95	0.95
Digestible Lys %	0.90	0.90	0.90	1.02	1.02	1.02	1.15	1.15	1.15	1.28	1.28	1.28
Digestible Thr %	0.60	0.60	0.60	0.69	0.69	0.69	0.77	0.77	0.77	0.86	0.86	0.86
Digestible Try %	0.22	0.18	0.18	0.21	0.21	0.21	0.24	0.24	0.24	0.27	0.27	0.27
Digestible Leu %	1.47	1.33	1.34	1.45	1.48	1.47	1.60	1.62	1.61	1.74	1.77	1.75
Digestible Val %	0.78	0.67	0.67	0.77	0.77	0.77	0.86	0.86	0.86	0.96	0.96	0.96
Digestible Arg %	1.15	0.96	0.96	1.14	1.13	1.13	1.30	1.29	1.29	1.47	1.45	1.46

Choline (ppm)	789	789	789	789	789	789	789	789	789	789	788	789
Chloride %	0.27	0.27	0.27	0.26	0.26	0.26	0.25	0.25	0.25	0.24	0.24	0.24
Sodium %	0.19	0.16	0.16	0.16	0.16	0.16	0.16	0.16	0.16	0.16	0.16	0.16
ME/CP (kcal/kg/%)	136.3	169.6	184.1	135.8	148.6	161.5	120.8	132.3	143.8	108.9	119.1	129.5

[1] Amino acids in the 100% diet were at the higher recommended levels of digestible amino acid (lysine, TSAA, and threonine).

[2] Premix did not contain riboflavin and provided the following per kilogram of finished diet: retinyl acetate, 2.654 μg; cholecalciferol, 110 μg; DL-α-tocopherol acetate, 9.9 mg; menadione, 0.9 mg; vitamin B12, 0.01 mg; folic acid, 0.6 μg; choline, 379 mg; D-pantothenic acid, 8.8 mg; niacin, 33 mg; thiamine, 1.0 mg; D-biotin, 0.1 mg; pyridoxine, 0.9 mg; ethoxyquin, 28 mg; manganese, 55 mg; zinc, 50 mg; iron, 28 mg; copper, 4 mg; iodine, 0.5 mg; selenium, 0.1 mg.

Table S 2 Feed ingredient and nutrient composition of 12 dietary treatments with factorial combinations of 4 levels of digestible amino acid and 3 levels of apparent metabolizable energy during the grower (d10-24) feeding phase

Parameter	Treatment											
AA (%)[1]	70	70	70	80	80	80	90	90	90	100	100	100
AME (%)	84	92	100	84	92	100	84	92	100	84	92	100
Yellow Corn %	62.18	72.39	74.46	62.24	69.27	68.45	57.33	65.86	61.93	53.92	61.10	55.41
Soybean Meal %	25.00	19.17	18.82	24.90	23.73	23.86	30.14	28.71	29.37	35.13	33.93	34.88
Soybean oil %	0.00	0.50	2.65	0.00	0.50	3.62	0.50	0.50	4.66	0.50	0.94	5.69
DL-Methionine %	0.17	0.20	0.20	0.26	0.25	0.25	0.30	0.29	0.30	0.34	0.34	0.34
L-Lysine HCl %	0.06	0.22	0.23	0.21	0.23	0.22	0.20	0.22	0.21	0.19	0.21	0.19
L-Threonine %	0.00	0.07	0.07	0.08	0.08	0.08	0.09	0.09	0.09	0.09	0.09	0.09
Ronozyme %	0.02	0.02	0.02	0.02	0.02	0.02	0.02	0.02	0.02	0.02	0.02	0.02
Dicalcium Phosphate %	1.51	1.51	1.51	1.51	1.50	1.50	1.49	1.48	1.48	1.48	1.46	1.47
Limestone %	1.36	1.38	1.38	1.35	1.36	1.36	1.33	1.35	1.34	1.32	1.33	1.32
Salt %	0.40	0.33	0.33	0.33	0.33	0.33	0.33	0.33	0.33	0.33	0.32	0.33
Premix[2] %	0.25	0.25	0.25	0.25	0.25	0.25	0.25	0.25	0.25	0.25	0.25	0.25
Choline chloride %	0.05	0.08	0.08	0.06	0.06	0.06	0.03	0.03	0.03	0.00	0.00	0.00
Sand %	9.00	3.88	0.00	8.80	2.43	0.00	7.99	0.88	0.00	6.44	0.00	0.00
Feed price ($/kg)	0.23	0.24	0.25	0.24	0.25	0.27	0.25	0.26	0.28	0.26	0.27	0.30
Calculated composition												
CP %	16.81	15.09	15.09	17.02	17.03	17.03	19.13	19.14	19.13	21.23	21.25	21.24
Ca %	0.87	0.87	0.87	0.87	0.87	0.87	0.87	0.87	0.87	0.87	0.87	0.87
Available *P* %	0.44	0.44	0.43	0.43	0.43	0.44	0.44	0.44	0.44	0.43	0.43	0.43
M.E. (kcal/kg)	2611	2859	3108	2611	2859	3108	2611	2859	3108	2611	2859	3108
Digestible Met %	0.4	0.42	0.42	0.49	0.48	0.49	0.55	0.55	0.55	0.62	0.62	0.62
Digestible TSAA %	0.61	0.61	0.61	0.7	0.7	0.7	0.78	0.78	0.78	0.87	0.87	0.87
Digestible Lys %	0.8	0.8	0.81	0.92	0.92	0.92	1.03	1.04	1.03	1.15	1.15	1.15
Digestible Thr %	0.54	0.54	0.54	0.62	0.62	0.62	0.69	0.69	0.69	0.77	0.77	0.77
Digestible Try %	0.19	0.16	0.16	0.19	0.19	0.19	0.22	0.22	0.22	0.25	0.24	0.24
Digestible Leu %	1.35	1.25	1.26	1.35	1.37	1.37	1.48	1.51	1.49	1.61	1.63	1.62
Digestible Val %	0.7	0.62	0.62	0.7	0.7	0.7	0.78	0.78	0.78	0.87	0.87	0.87
Digestible Arg %	1.02	0.86	0.86	1.01	1	1	1.16	1.15	1.15	1.31	1.3	1.31

Choline (ppm)	729	729	729	729	729	729	729	729	729	730	729	730
Chloride %	0.27	0.27	0.27	0.26	0.26	0.26	0.25	0.25	0.25	0.24	0.24	0.24
Sodium %	0.19	0.16	0.16	0.16	0.16	0.16	0.16	0.16	0.16	0.16	0.16	0.16
ME/CP (kcal/kg/%)	155.3	189.5	206.0	153.4	167.9	182.5	136.5	149.4	162.5	123.0	134.5	146.3

[1] Amino acids in the 100% diet were at the higher recommended levels of digestible amino acid (lysine, TSAA, and threonine).

[2] Premix did not contain riboflavin and provided the following per kilogram of finished diet: retinyl acetate, 2.654 μg; cholecalciferol, 110 μg; DL-α-tocopherol acetate, 9.9 mg; menadione, 0.9 mg; vitamin B12, 0.01 mg; folic acid, 0.6 μg; choline, 379 mg; D-pantothenic acid, 8.8 mg; niacin, 33 mg; thiamine, 1.0 mg; D-biotin, 0.1 mg; pyridoxine, 0.9 mg; ethoxyquin, 28 mg; manganese, 55 mg; zinc, 50 mg; iron, 28 mg; copper, 4 mg; iodine, 0.5 mg; selenium, 0.1 mg.

Table S 3 Feed ingredient and nutrient composition of 12 dietary treatments with factorial combinations of 4 levels of digestible amino acid and 3 levels of apparent metabolizable energy during the finisher (d 24-41) feeding phase

Parameter	Treatment											
AA (%)[1]	70	70	70	80	80	80	90	90	90	100	100	100
AME (%)	84	92	100	84	92	100	84	92	100	84	92	100
Yellow Corn %	67.99	75.30	73.54	64.37	72.78	68.94	61.57	69.16	63.72	58.76	63.94	58.50
Soybean Meal %	19.96	18.67	18.98	23.28	22.36	22.78	27.38	26.55	27.15	31.48	30.92	31.51
Soybean oil %	0.00	0.50	4.01	0.50	0.50	4.77	0.50	0.78	5.61	0.50	1.63	6.45
DL-Methionine %	0.16	0.15	0.15	0.21	0.20	0.20	0.25	0.24	0.25	0.30	0.29	0.30
L-Lysine HCl %	0.09	0.11	0.11	0.12	0.13	0.13	0.13	0.13	0.13	0.13	0.14	0.13
L-Threonine %	0.00	0.01	0.01	0.03	0.02	0.03	0.04	0.03	0.04	0.05	0.05	0.05
Ronozyme %	0.02	0.02	0.02	0.02	0.02	0.02	0.02	0.02	0.02	0.02	0.02	0.02
Dicalcium Phosphate %	1.28	1.26	1.27	1.27	1.25	1.26	1.25	1.24	1.25	1.24	1.23	1.24
Limestone %	1.26	1.27	1.27	1.25	1.26	1.25	1.23	1.25	1.24	1.22	1.23	1.22
Salt %	0.33	0.33	0.33	0.33	0.33	0.33	0.33	0.33	0.33	0.33	0.33	0.33
Premix[2] %	0.25	0.25	0.25	0.25	0.25	0.25	0.25	0.25	0.25	0.25	0.25	0.25
Choline chloride %	0.06	0.06	0.06	0.05	0.04	0.04	0.02	0.02	0.02	0.00	0.00	0.00
Sand %	8.60	2.06	0.00	8.32	0.86	0.00	7.02	0.00	0.00	5.72	0.00	0.00
Feed price ($/kg)	0.22	0.23	0.25	0.23	0.24	0.26	0.24	0.25	0.28	0.25	0.26	0.29
Calculated composition												
CP %	14.92	14.90	14.91	16.28	16.50	16.40	18.03	18.23	18.09	19.78	19.92	19.77
Ca %	0.78	0.78	0.78	0.78	0.78	0.78	0.78	0.78	0.78	0.78	0.78	0.78
Available *P* %	0.39	0.39	0.39	0.39	0.39	0.39	0.39	0.39	0.39	0.39	0.39	0.39
M.E. (kcal/kg)	2689	2944	3200	2688	2944	3200	2688	2944	3200	2688	2944	3200
Digestible Met %	0.37	0.37	0.37	0.44	0.43	0.43	0.50	0.49	0.50	0.56	0.56	0.56
Digestible TSAA %	0.56	0.56	0.56	0.64	0.64	0.64	0.72	0.72	0.72	0.80	0.80	0.80
Digestible Lys %	0.71	0.71	0.71	0.82	0.82	0.82	0.92	0.92	0.92	1.02	1.02	1.02
Digestible Thr %	0.48	0.48	0.48	0.54	0.54	0.54	0.61	0.61	0.61	0.68	0.68	0.68
Digestible Try %	0.17	0.16	0.16	0.18	0.18	0.18	0.20	0.21	0.20	0.23	0.23	0.23
Digestible Leu %	1.24	1.26	1.25	1.31	1.36	1.34	1.42	1.46	1.44	1.53	1.56	1.53
Digestible Val %	0.62	0.62	0.62	0.67	0.68	0.68	0.74	0.75	0.75	0.81	0.82	0.81
Digestible Arg %	0.87	0.86	0.86	0.97	0.97	0.97	1.09	1.09	1.09	1.21	1.21	1.21

Choline (ppm)	693	693	693	693	693	693	693	693	693	693	696	693
Chloride %	0.24	0.24	0.24	0.24	0.24	0.24	0.23	0.23	0.23	0.23	0.23	0.23
Sodium %	0.16	0.16	0.16	0.16	0.16	0.16	0.16	0.16	0.16	0.16	0.16	0.16
ME/CP (kcal/kg/%)	180.2	197.6	214.6	165.1	178.4	195.1	149.1	161.5	176.9	135.9	147.8	161.9

[1] Amino acids in the 100% diet were at the higher recommended levels of digestible amino acid (lysine, TSAA, and threonine).

[2] Premix did not contain riboflavin and provided the following per kilogram of finished diet: retinyl acetate, 2.654 μg; cholecalciferol, 110 μg; DL-α-tocopherol acetate, 9.9 mg; menadione, 0.9 mg; vitamin B12, 0.01 mg; folic acid, 0.6 μg; choline, 379 mg; D-pantothenic acid, 8.8 mg; niacin, 33 mg; thiamine, 1.0 mg; D-biotin, 0.1 mg; pyridoxine, 0.9 mg; ethoxyquin, 28 mg; manganese, 55 mg; zinc, 50 mg; iron, 28 mg; copper, 4 mg; iodine, 0.5 mg; selenium, 0.1 mg.

Table S 4 Feed ingredient and nutrient composition of 12 dietary treatments with factorial combinations of 4 levels of digestible amino acid and 3 levels of apparent metabolizable energy during the withdrawal (d 41-64) feeding phase

Parameter	Treatment											
AA (%)[1]	70	70	70	80	80	80	90	90	90	100	100	100
AME (%)	84	92	100	84	92	100	84	92	100	84	92	100
Yellow Corn	68.69	77.60	73.34	67.61	76.08	70.69	64.94	71.21	65.74	62.28	66.25	60.78
Soybean Meal	19.84	18.27	19.02	21.42	20.49	21.08	25.32	24.63	25.23	29.22	28.78	29.38
Soybean oil	0.00	0.00	4.37	0.00	0.00	4.83	0.00	0.77	5.63	0.00	1.57	6.43
DL-Methionine	0.12	0.11	0.12	0.18	0.17	0.18	0.22	0.21	0.22	0.26	0.26	0.27
L-Lysine HCl	0.04	0.07	0.05	0.11	0.12	0.12	0.12	0.12	0.12	0.12	0.12	0.12
L-Threonine	0.00	0.00	0.00	0.02	0.01	0.02	0.03	0.02	0.03	0.04	0.03	0.04
Ronozyme	0.02	0.02	0.02	0.02	0.02	0.02	0.02	0.02	0.02	0.02	0.02	0.02
Dicalcium Phosphate	1.22	1.21	1.21	1.21	1.20	1.21	1.20	1.19	1.20	1.19	1.18	1.19
Limestone	1.24	1.25	1.24	1.23	1.24	1.24	1.22	1.23	1.22	1.21	1.21	1.20
Salt	0.33	0.33	0.33	0.33	0.33	0.33	0.33	0.33	0.33	0.33	0.33	0.33
Premix[2]	0.25	0.25	0.25	0.25	0.25	0.25	0.25	0.25	0.25	0.25	0.25	0.25
Choline chloride	0.05	0.05	0.05	0.04	0.04	0.04	0.02	0.02	0.02	0.00	0.00	0.00
Sand	8.20	0.84	0.000.00	7.57	0.05	0.00.	6.33	0.00	0.00	5.10	0.00	0.00
Feed price ($/kg)	0.22	0.23	0.25	0.23	0.23	0.26	0.23	0.25	0.27	0.24	0.26	0.29
Calculated composition												
CP, %	14.84	14.83	14.83	15.62	15.85	15.70	17.28	17.45	17.31	18.95	19.05	18.91
Ca, %	0.76	0.76	0.76	0.76	0.76	0.76	0.76	0.76	0.76	0.76	0.76	0.76
Available *P*, %	0.38	0.38	0.38	0.38	0.38	0.38	0.38	0.38	0.38	0.38	0.38	0.38
M.E. (kcal/kg)	2709	2967	3225	2709	2967	3225	2709	2967	3225	2709	2967	3225
Digestible Met, %	0.33	0.33	0.33	0.40	0.40	0.40	0.46	0.46	0.46	0.52	0.52	0.52
Digestible TSAA, %	0.53	0.53	0.53	0.60	0.60	0.60	0.67	0.67	0.67	0.75	0.75	0.75
Digestible Lys, %	0.67	0.67	0.67	0.77	0.77	0.77	0.86	0.86	0.86	0.96	0.96	0.96
Digestible Thr, %	0.47	0.47	0.47	0.51	0.51	0.51	0.58	0.58	0.58	0.64	0.64	0.64
Digestible Try	0.17	0.16	0.16	0.17	0.17	0.17	0.19	0.20	0.19	0.22	0.22	0.22
Digestible Leu	1.24	1.26	1.25	1.28	1.32	1.30	1.39	1.42	1.39	1.49	1.51	1.48
Digestible Val	0.62	0.62	0.62	0.65	0.65	0.65	0.71	0.72	0.71	0.78	0.79	0.78
Digestible Arg	0.87	0.85	0.86	0.92	0.92	0.92	1.04	1.03	1.03	1.15	1.15	1.15

Choline (ppm)	670	670	670	670	670	670	670	670	670	671	674	671
Chloride, %	0.22	0.23	0.23	0.24	0.23	0.24	0.23	0.23	0.23	0.22	0.22	0.22
Sodium, %	0.16	0.16	0.16	0.16	0.16	0.16	0.16	0.16	0.16	0.16	0.16	0.16
ME/CP (kcal/kg/%)	182.5	200.1	217.5	173.4	187.2	205.4	156.8	170.0	186.3	143.0	155.7	170.5

[1] Amino acids in the 100% diet were at the higher recommended levels of digestible amino acid (lysine, TSAA, and threonine).

[2]Premix did not contain riboflavin and provided the following per kilogram of finished diet: retinyl acetate, 2.654 μg; cholecalciferol, 110 μg; DL-α-tocopherol acetate, 9.9 mg; menadione, 0.9 mg; vitamin B12, 0.01 mg; folic acid, 0.6 μg; choline, 379 mg; D-pantothenic acid, 8.8 mg; niacin, 33 mg; thiamine, 1.0 mg; D-biotin, 0.1 mg; pyridoxine, 0.9 mg; ethoxyquin, 28 mg; manganese, 55 mg; zinc, 50 mg; iron, 28 mg; copper, 4 mg; iodine, 0.5 mg; selenium, 0.1 mg.
